# Molecularly Imprinted Polymer-Based Microfluidic Systems for Point-of-Care Applications

**DOI:** 10.3390/mi10110766

**Published:** 2019-11-11

**Authors:** Yeşeren Saylan, Adil Denizli

**Affiliations:** Department of Chemistry, Hacettepe University, Ankara 06800, Turkey; yeseren@hacettepe.edu.tr

**Keywords:** microfluidic, molecular imprinting, point-of-care, polymers

## Abstract

Fast progress has been witnessed in the field of microfluidic systems and allowed outstanding approaches to portable, disposable, low-cost, and easy-to-operate platforms especially for monitoring health status and point-of-care applications. For this purpose, molecularly imprinted polymer (MIP)-based microfluidics systems can be synthesized using desired templates to create specific and selective cavities for interaction. This technique guarantees a wide range of versatility to imprint diverse sets of biomolecules with different structures, sizes, and physical and chemical features. Owing to their physical and chemical robustness, cost-friendliness, high stability, and reusability, MIP-based microfluidics systems have become very attractive modalities. This review is structured according to the principles of MIPs and microfluidic systems, the integration of MIPs with microfluidic systems, the latest strategies and uses for point-of-care applications and, finally, conclusions and future perspectives.

## 1. Introduction

Selective and sensitive biomolecule detection plays an important role in many systems [1]. Although natural recognition elements have high affinity to their target molecules, they have limited application due to sensitive features, including low durability and stability at high pressure and temperature in different media [2]. In recent times, under the category of supramolecular chemistry, the molecular imprinting technique has been offered as a means to overcome most of these drawbacks. This technique was first reported in the 1970s [3] and has since been adopted by many scientists, as evidenced by the increase in studies from all over the world. It is generally and principally based on the selective recognition of template molecules [4]. Molecularly imprinted polymers (MIPs) can also be synthesized using various functional monomers, template molecules, crosslinkers, initiators, and solvent compositions [5,6,7,8,9]. The MIPs have also a lot of benefits, such as easy preparation, cost-friendliness, and high stability, selectivity, and affinity toward the template molecules. The quality of the MIPs can be changed with different combinations of experimental conditions and interaction mechanisms [10,11,12]. 

Microfluidics is the science and technology of systems that process small amounts of fluids (10^−9^–10^−18^ L), employing channels with dimensions of tens to hundreds of micrometers [13]. They have a huge potential to impact different subject areas, from biological analysis to information technology. Microfluidic systems possess a number of useful advantages, such as the use small of quantities of samples and detection with high resolution and sensitivity, short analysis time, low cost, and small footprints [14,15]. Microfluidic systems are also small in size and the properties of fluids in microchannels are less visible. They especially present fundamentally novel abilities in the control of biomolecule concentrations [16]. In many ways, the basic properties of microfluidic systems allow for their natural adaption information point-of-care diagnostics devices, such as for diagnostic tests performed near the patient without the need for a clinical laboratory and allowing for the low consumption of reagents and sample, miniaturization of devices, and fast turnaround time for analysis [17,18]. Due to the need to detect important biomolecules, the applications of molecular screening in diagnostics, as well as in food and environmental fields are growing fast [19]. Biomolecule detection in several samples is frequently carried out in laboratories employing commercial techniques, including immunoassays and chromatographic techniques that are laborious, time-consuming, expensive, and require rigorous conditions and specialized staff [20]. Thus, the interest and need for the fabrication of cheaper, faster, user-friendly, and reusable detection platforms has been increasing. The recognition elements can be bound to the biomolecules that are immobilized on a signal transducer in these platforms. The binding interactions can be translated by readout methods into a concentration-dependent signal [21].

In this review, the fundamentals of MIPs and microfluidic systems, the integration of MIPs with microfluidic systems, and their recent uses in the applications of point-of-care applications are extensively discussed. The conclusions and future perspectives are summarized at the end of the review.

## 2. Fundamentals of Molecularly Imprinted Polymers (MIPs)

**Figure 1:**
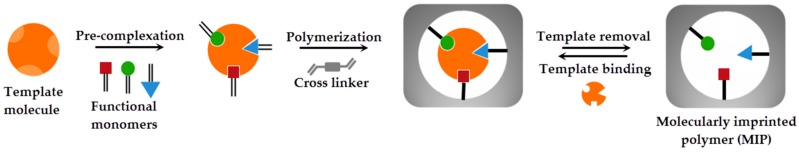
The steps of the molecularly imprinted polymer (MIP) preparation.

The template molecules, functional monomers, crosslinkers, initiators, and solvents are essential and key elements for the polymerization of MIPs. At first, the template molecule interacts with the functional monomer to acquire pre-complex structures and the pre-complex is then polymerized in the presence of a crosslinker to obtain a polymeric matrix [22]. After template molecule removal, template-specific binding cavities are formed in the polymeric matrix and the MIP is ready for interaction (Figure 1).

Template molecule selection is the most significant step in MIP preparation. In this step, crosslinking functional monomers are surrounded by the selected template molecule with specific interactions. After the polymerization, the template molecule is removed by suitable eluents from the polymeric matrix to obtain MIPs that have specific template molecule-binding sites [23,24]. The other important part is functional monomer selection, which is a crucial step to prepare the optimum combination of template molecule and functional monomer. On the other hand, it only increases the complexity of the chemical environment and may end up in disruptions [25,26]. Additionally, the crosslinker makes the polymeric matrix chain bind to another chain. It plays crucial functions to produce a rigid polymeric matrix and also comprises recognition groups [27]. Another important element is the initiator, that effects polymer production by linking a large number of monomers. The initiator should be selected due to the polymerization type and template molecule [28]. The last element is a solvent that makes a more expanded polymeric matrix chain, or the chains stay close to each other. It has a vital role as a porogen maker that checks the morphology and porosity of the polymeric matrix [29]. Thus, MIPs can be synthesized by using functional monomers and template molecules in the presence of appropriate crosslinkers, initiators, and solvents. The template-specific cavities are obtained with a certain size, shape, and chemical and physical effectivity at the end of the polymerization [30]. In the literature, there are several studies about new recognition elements such as MIPs, phages, aptamers, and affibodies. They are preferred to be use for polymerization instead of classical recognition elements (enzymes, antibodies, nucleic acids, and whole cells) due to their ability to mimic natural recognition entities and providing a versatile platform to achieve the desirable functionality for various point-of-care applications [31,32,33].

## 3. Principle of Microfluidic Systems

**Figure 2:**
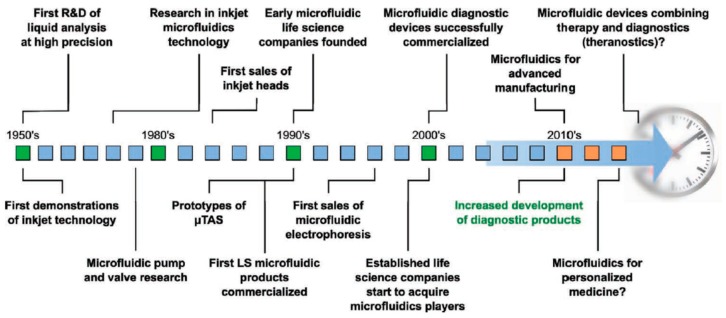
The timeline of the microfluidic technology evolution. Republished with permission from Gervais et al. [38].

The principle of microfluidic systems depends on the science of studying the behavior of the flow of fluid through and around structures at the microscale level. Microfluidic systems have to test themselves against the classical systems and provide opportunities such as high sensitivity, low cost, short time, and lower consumption of laboratory space. In addition, scaling effects guide phenomena and allow new applications that are not accessible to classical platforms, including a high grade of parallelization, laminar flow with liquid gradients down to single-cell-length scales, high-speed serial processing, and structures the size of a cell. The amount of reagent consumption can be minimized significantly by scaling down the assay volume and also by reducing the footprint of each test. With decreasing length scales, capillary forces become increasingly dominant over volume forces and enable passive liquid actuation used in the capillary test strips. Another effect is the onset of laminar flow at low Reynolds numbers in microchannels that allows the creation of well-defined and stable liquid–liquid interfaces down to cellular dimensions [34,35,36,37]. As depicted in Figure 2, this microfluidic system technology has a long history from research to diagnostics used in hospitals. The evolution started at around the 1950s, and still continues to grow to supply certain needs [38].

## 4. Integrations of MIPs with Microfluidic Systems

Microfluidic systems and MIPs have been integrated and extensively utilized in point-of-care applications, thanks to significant advantages such as the need for low sample volume, integrality selectivity, and sensitivity. Practicality gains attention in the generation of integrated systems that are self-contained, automated, and rapid [39,40]. Furthermore, microfluidic systems have been frequently used for the nanoparticle and nanofilm immobilization. These nanomaterials have exclusively been used in MIP-based microfluidic systems for different applications, such as quartz crystal microbalances, surface plasmon resonance, and colorimetric and fluorescence sensors [41,42].

## 5. Latest Strategies of MIP-Based Microfluidic Systems

Recently, several technologies of molecular imprinting techniques have earned prompt development according to the continuous use and improvement in traditional polymerization and modification techniques. Moreover, diversified MIP-based microfluidic systems with excellent performances have been prepared for exciting and universal applications [43].

### 5.1. Polymers

**Figure 3:**
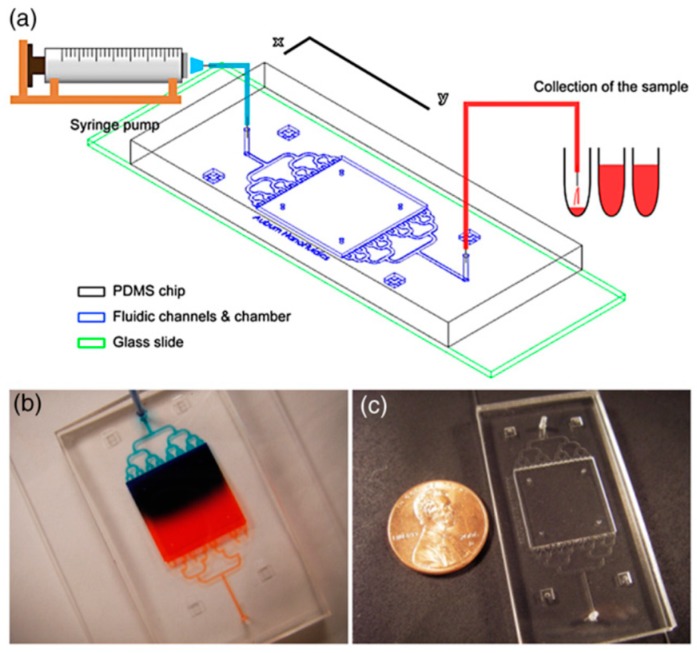
The microfluidic system design. Republished with permission from Ali et al. [45].

MIPs have shown high performance in several applications, generally exceeding the conventional methods and providing a more cost-effective approach [44]. For instance, Ali et al. prepared MIP-based hydrogels for the release of ketotifen fumarate that were employed in therapeutic contact lenses. They carried out in vitro drug release studies from contact lenses within a microfluidic system that simulated tear volume and tear composition of an eye. As shown in Figure 3, the MIP-based hydrogel was placed in the microfluidic chamber between the four posts and drug release was quantified within artificial lacrimal fluid flow rates. They reported that this contact lens has a suspended release of the drug compared to less functionalized systems. In addition, this MIP-based contact lens showed Fickian release kinetics, with diffusion coefficients ranging from 4.04 × 10^−9^ to 5.57 × 10^−10^ cm^2^/s [45]. 

Shiraki et al. also synthesized a MIP-based hydrogel with a microfluidic system to detect bisphenol A that has a high ability to interrupt the endocrine system. They used cyclodextrin as a ligand and polymerized using a fluorescence microscope. They showed that this MIP-based hydrogel had an ultra-fast shrinkage in response to bisphenol A and, also, that the flow rate of the microchannel was adjusted by the shrinking of the hydrogels [46].

**Figure 4:**
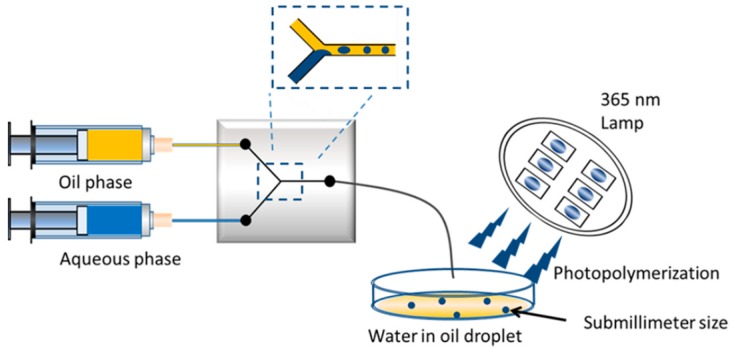
The submillimeter sized MIP-based microgels preparation. Republished with permission from Takimoto et al. [47].

Takimoto et al. prepared submillimeter-sized MIP-based microgels using polymerization of water-soluble monomers with a photoinitiator in water-in-oil droplets produced by the microchannel (Figure 4). After the optimization of suitable surfactants, surfactant concentration, and flow rate selection, the microgel size was managed by the oil phase flow rate for human serum albumin recognition. Furthermore, they reported the submillimeter-sized MIP-based microgels exhibited a high selective and affinity binding toward human serum albumin [47].

Ren et al. covered glass with bacteria that was pushed into another glass covered with polydimethylsiloxane. The polydimethylsiloxane was hardened, and the bacteria were eluted to produce a textured surface whose indentations capture the same type of bacteria when mixture flows over it. They found that the selectivity between the Gram-negative and Gram-positive bacteria groups was stronger than the bacteria in the same group. Furthermore, some selectivity was also detected between the closely related species in the same Gram bacteria groups [48].

**Figure 5:**
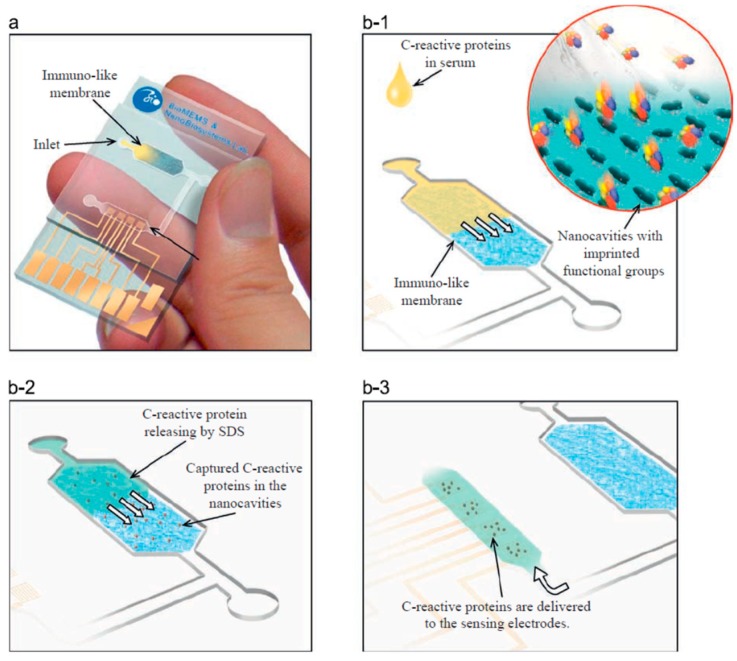
The illustration of the MIP-based nanocavities (immuno-like membrane in microfluidic system (**a**); loading of serum samples into the microfluidic system and capturing C-reactive protein from serum samples (**b-1**); loading of SDS and releasing of C-reactive protein from the immuno-like membrane (**b-2**); delivery of SDS with C-reactive protein to the electrodes (**b-3**)). Republished with permission from Hong et al. [49].

Hong et al. presented an immune-like membrane for separation and sensation C-reactive protein in serum samples employing the MIP-based nanocavities. They enhanced the performance of separation by an alignment of the C-reactive protein. They synthesized aligned the MIP-based nanocavities and integrated with microfluidic systems as point-of-care applications (Figure 5). They demonstrated that the adhesion forces of the MIP-based nanocavities on the immuno-like membranes were comparable to the interaction forces between C-reactive protein and antibodies [49].

Kellens et al. demonstrated a microfluidic system in conjunction based on functionalized diamond substrates. They claimed that this strategy was low cost, simple, and efficient for testosterone detection in urine, buffer, and saliva samples. They also adapted this microfluidic system to a sensor using electrochemical impedance spectroscopy and obtained a low limit of detection value (0.5 nM) under a wide range of testosterone concentrations [50].

### 5.2. Sensors

**Figure 6:**
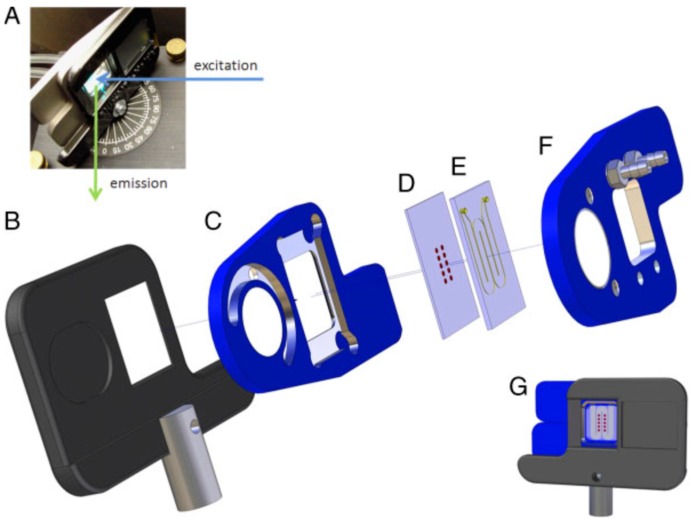
The microfluidic system and holder for fluorescence measurements (excitation and emission light beams in a spectrofluorometer (**A**); holder for the microfluidic system (**B**); magnetically connected holder with window (**C**) and (**F**); substrate on MIPs (**D**); microstructured meander for fluid transport (**E**); arranged setup with all components (**G**)). Republished with permission from Thaler et al. [52].

Due to their insufficient lower limits of detection and their lower analytical specificity, as compared to antibodies, the quantification and detection of some clinically relevant biomolecules by MIP-based sensors has been gaining importance and studied extensively in recent years [51]. For example, Harz et al. described a study on an immobilized MIPs with a microfluidic system for fluorescence detection of dansyl-L-phenylalanine. They presented the immobilization of the polymers on quartz surfaces to produce homogenous films and their uses in a spectrofluorometer. As depicted in Figure 6, the excitation and emission lights were represented, with holder, excitation angle, and easy adjustment of position at the system also demonstrated. They showed more sensitive detection of dansyl-L-phenylalanine (fifty times) compared to without MIPs [52].

Sharma et al. designed a MIP-based receptor that has cavities to detect an autism biomarker (oxytocin nonapeptide). They deposited this MIP-based receptor by electropolymerization on a gold film electrode in an electrochemical microfluidic system. They performed the kinetic studies in a different biomarker concentration range (from 0.06 to 1 mM) with a detection limit of 60 μM. They also carried out sensitivity analysis of the microfluidic system in both synthetic serum and aqueous samples, and reported that this MIP-based microfluidic system was selective to common interferences, including oxytocin analogs and potential metabolites [53].

Liu et al. established an electrochemical detection platform by integrating MIPs with a microfluidic system and utilized this for therapeutic drug detection. They characterized the working electrode via electrochemical impedance spectroscopy and cyclic voltammetry and tested the linearity of the method in the range of 5 × 10^−6^ to 4 × 10^−4^ M. By contrast, the linearity of gate effect was 2 × 10^−11^ to 4 × 10^−9^ M with a low detection limit (8 × 10^−12^ M) which is suitable for clinical assays. They also applied this to monitor drug concentrations in rabbit plasma over a day [54].

Weng et al. designed a microfluidic system to detect morphine employing the MIP-based electrochemical sensing principle. They integrated a polydimethylsiloxane microchannel, a peristaltic micropump, microvalves, and sensing microelectrodes to prepare a MIP-based microfluidic system for morphine sensing. The morphine samples were transported to the electrode using the automatic peristaltic micropump. They showed that the sensitivity of the MIP-based microfluidic system is 0.3 μM in detecting morphine concentrations ranging from 0.01 to 0.2 mM [55].

**Figure 7:**
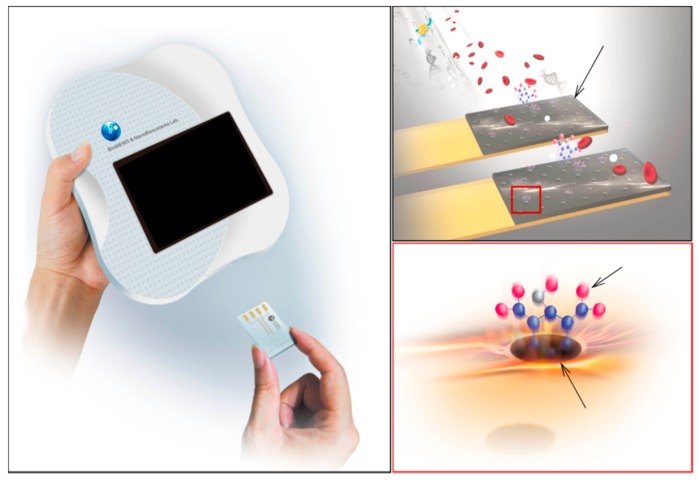
The propofol sensing system. Republished with permission from Hong et al. [56].

**Figure 8:**
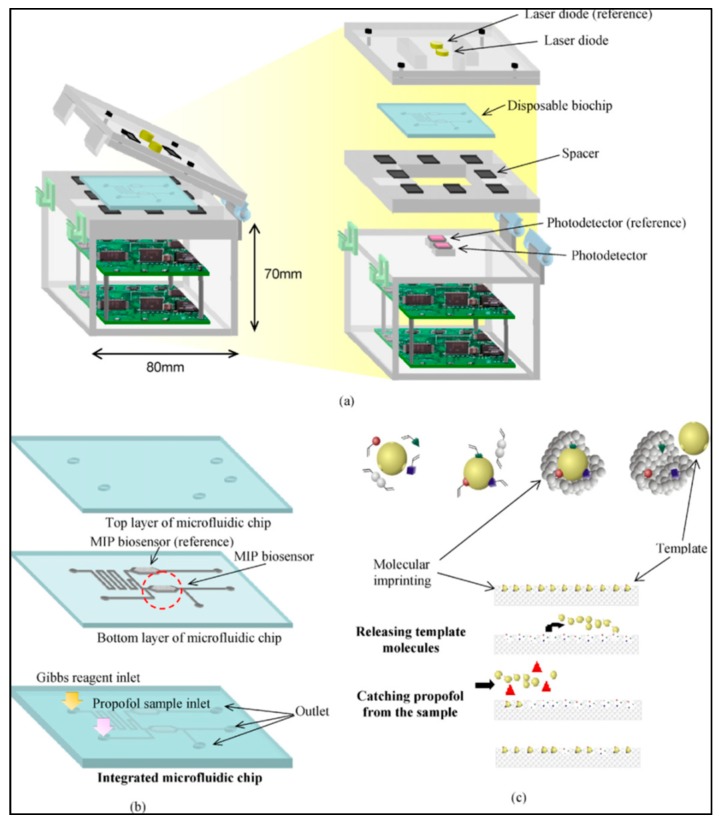
The microfluidic system with MIPs for propofol detection. Republished with permission from Hong et al. [57].

Hong et al. proposed a handheld analyzer with a disposable system for detection of anesthetic propofol in total intravenous anesthesia with a target controlled in a fusion system for detection in plasma samples. Their system is based on the conduction of MIPs and electrical detection (Figure 7). They employed this for detecting blood propofol concentrations in hospitals to compare with other conventional methods (high-performance liquid chromatography and ion mobility spectrometry). They revealed that the response time of the system was very short (25 s) and the detection limit was 0.1 μg/mL with a range of 0.1–30 μg/mL [56]. The same research group also presented a disposable microfluidic system for optical detection of propofol (Figure 8). They integrated MIPs into the microfluidic system to be used for propofol detection optically at 655 nm wavelength after the reaction of propofol with color reagent and obtained the limit of detection value as 0.25 ppm in a range from 0.25 to 10 ppm [57]. 

Dejous et al. introduced an electronic nose or tongue for data processing in complex media such as biomarkers in breath or liquid. They exemplified with a versatile acoustic wave transducer that was modified with MIPs to detect adenosine-51-monophosphate. They described the thin film coating and then demonstrated the static measurements with electrical and scanning electron microscopy characterization after each step of the process [58].

### 5.3. Papers

The emerging paper-based microfluidics systems show more seductive benefits with more useful components being integrated into a platform in several applications [59,60,61]. The papers have been adopted as attractive detection platforms due to their incomparable properties, such as low sample consumption and low cost and, also, pump-free transportation [62]. 

**Figure 9:**
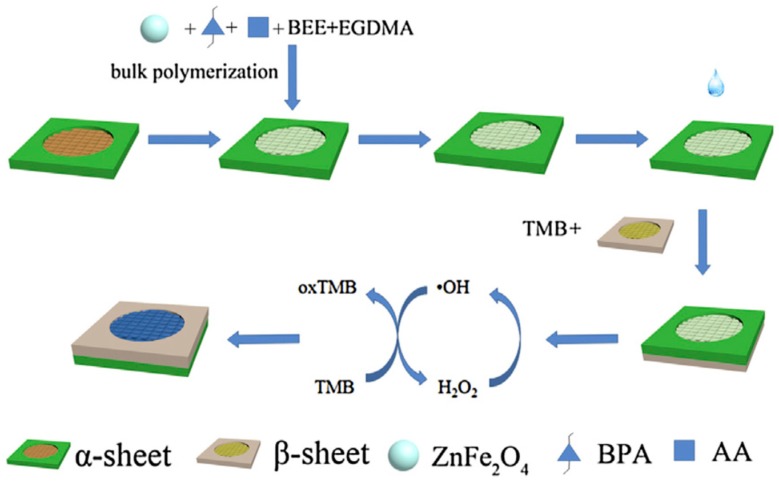
The paper-based colorimetric sensor fabrication steps. Republished with permission from Kong et al. [63].

Kong et al. developed a paper-based colorimetric microfluidic system to detect bisphenol A using magnetic nanoparticle peroxidase activity and the MIP-based membrane adsorption capacities. As depicted in Figure 9, this system was fabricated via MIP-based membrane immobilization. They employed the isotherm models of MIP-based membranes to determine the interaction and evaluated maximum adsorption capacity. They found the grey intensity to be comparable for the bisphenol A concentrations in the range of 10–1000 nM with a detection limit of 6.18 nM [63]. The same research group also constructed a paper-based system utilizing multiplate nanoflowers and MIP-based membranes for amino acid detection. They reported this system selectively detected the analytes as well as decreased the electron loss via blocking the reduction reaction between electrons and products. This device showed the detection limits toward two different amino acids to be as low as 9.6 and 24 pM. They also pointed out that nanoflowers showed superior signals associated with nanospheres, nanosheets, and nanorod platforms [64].

**Figure 10:**
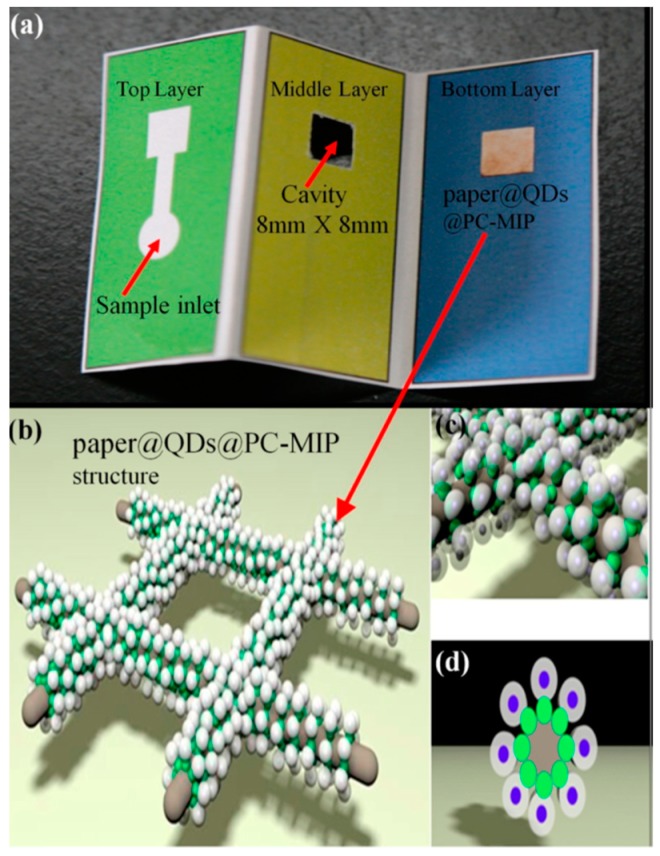
The structure of the origami paper-based microfluidic system. Republished with permission from Li et al. [65].

Li et al. developed an origami paper-based microfluidic system employing quantum dots related to MIPs for the sensitive and selective recognition of phycocyanin. Figure 10 schematically illustrates the structure and the sensing principle of a three-dimensional origami paper-based microfluidic system. This system could realize the liquid phase of quantum dots combined with MIPs being transferred to the solid-phase paper base and achieved easy portability. They reported that their three-dimensional origami paper-based microfluidic system could detect phycocyanin in a dynamic range of 10−50 mg/L with a limit of detection of 2 mg/L [65]. The same research group also proposed an origami ion-imprinted microfluidic paper-based system to multiplex detect Cu^2+^ and Hg^2+^ ions. They activated the surfaces by grafting with quantum dots and carried out the formation of quantum dots-based ion-imprinted complex that led to fluorescence quenching. They reported that the Cu^2+^ ion-imprinted fluorescent platform exhibited linearity from 0.11 to 58.0 µg/L with a detection limit of 0.035 µg/L and the Hg^2+^ ion linear range is 0.26–34.0 µg/L with a detection limit of 0.056 µg/L [66].

Ge et al. also fabricated a paper-based microfluidic system using electropolymerization of MIPs in gold nanoparticle-modified paper. This was prepared through the growth of a gold nanoparticle layer on the surfaces of fibers in the paper working electrode. They developed a microfluidic origami system that consisted of an auxiliary pad encompassed by sample tabs to detect amino acid D-glutamic acid in a linear range from 1.2 to 125.0 nM with a low detection limit (0.2 nM) [67].

## 6. Conclusions and Future Perspectives

During recent years, the health status of people has become a major public concern with interest in disease detection growing all over the world. For example, air pollution possesses a significant threat and causes several diseases, such as oncologic, cardiovascular, neurodegenerative, and also chronic respiratory diseases. Conventional methods applied for diagnosis of these diseases usually comprises invasive and hazardous biopsy, mammography, endoscopy, blood tests, computed tomography, microbial culture tests, magnetic resonance imaging, ultrasonography, positron emission tomography, and/or X-ray imaging of organs. Less or more highly invasive methods not only represent some risks of dangerous adverse side effects, including surgical complications and internal burns, but they frequently dispirit patients from participating in preventive screening procedures. Moreover, conventional methods, despite offering a convenient way to analyze complex samples, also have some disadvantages and limitations. In addition, these methods are not tractable for routine and rapid assays due to the polar nature of biomolecules and certain complex sample pre-treatment before analysis. They also require sample handling and blood collection, with possible contamination and requirement of highly skilled personnel. They are often also time consuming and need expensive and sophisticated instrumentation. Hence, there is still a huge need for the development of proficient detection systems in order to pre-empt this problem in public health. In these circumstances, microfluidic systems are assumed to propose complementary tools to conventional methods, as devices devoted to biomolecules with a fast response time and convenience of usage. Among the systems under development, some aim to achieve high quality and precision of test results for diagnostic purposes and the detection of diseases in their early stages. In addition, MIP-based microfluidic systems offer robust, reusable, and highly selective and sensitive platforms to detect important biomolecules.

This review article aimed to discuss the principle of MIPs and the integration of MIPs with microfluidic systems. The recent studies of MIP-based microfluidic systems were extensively overviewed for different targets in point-of-care applications. They were prepared using different template molecules, functional monomers, crosslinkers, initiators, and solvents. Compared with conventional techniques, these MIP-based microfluidic systems have several advantages and show more promising applications for improving human health (Table 1).

## Figures and Tables

**Table 1:** Summary of the recent MIP-based microfluidic systems.

Material	Target	Advantages	Dynamic Range	Detection Limit	Reference
Hydrogel	Ketotifen fumarate	A potential determination of physiological release rates, matching local conditions to characterize drug delivery devices	4.04 × 10^−9^ to 5.57 × 10^−10^ cm^2^/s	–	[45]
Hydrogel	Bisphenol A	An ultra-fast shrinkage in response, adjusted flow rate by the shrinking of the hydrogels	120 μg/mL	–	[46]
Microgel	Human serum albumin	A high affinity, selectivity and stability	5 μM	–	[47]
Film	*Staphylococcus epidermidis*, *Staphylococcus aureus*, *Escherichia coli*, and *Klebsiella pneumoniae*	One sorting cycle to capture, release a pure bacterial strain, the dominant role of chemical recognition	10^9^ cells/mL	–	[48]
Membrane	C-reactive protein	A specific and cost-effective approach, catch specific proteins in complex	0–200 μg/mL	–	[49]
Microstructure	Testosterone	A low-cost, simple, robust, efficient, less time consuming	0.5–500 nM	0.5 nM	[50]
Fluorescence	Dansyl-L-phenylalanine	A high sensitivity and selectivity	1–100 μM	0.5 μM	[52]
Electrochemical	Oxytocin nonapeptide	A high sensitivity and selectivity	0.06–1 mM	60 μM	[53]
Electrochemical	Warfarin sodium	An accurate, reliable, interference-free, simple, low-cost	2 × 10^−11^ to 4 × 10^−9^ M	8 × 10^−12^ M	[54]
Electrochemical	Morphine	A precise and continuous measurement, compact in size, consumes fewer samples	0.01–0.2 mM	0.3 μM	[55]
Electrochemical	Propofol	A compact size, high selectivity, low cost, rapid response, single-step detection	0.1–30 μg/mL	0.1 μg/mL	[56]
Optical	Propofol	A disposable, high selectivity, low cost, rapid response, single-step detection	0.25–10 ppm	0.25 ppm	[57]
Film	Adenosine-51-monophosphate	A detection in real-time, low concentrations of nucleoside analogues, good stability	5–600 ppm	5 ppm	[58]
Magnetic nanoparticle	Bisphenol A	A highly reproducible response, good selectivity, excellent regeneration	10–1000 nM	6.18 nM	[63]
Nanoflowers	L-glutamic acid and L-cysteine	A selective, accurate, rapid, inexpensive, on-site monitoring	20 pM to 1000 nM and 50 pM to 800 nM	9.6 pM and 24 pM	[64]
Quantum dot	Phycocyanin	A robust, facile route to detection, portability, disposability, low cost, user-friendly protocol	10−50 mg/L	2 mg/L	[65]
Quantum dot	Cu^2+^ and Hg^2+^ ions	A novel, simple, convenient analysis, cost-effective, portable	0.11 to 58.0 µg/L (Cu^2+^) and 0.26–34.0 µg/L (Hg^2+^)	0.035 µg/L (Cu^2+^) and 0.056 µg/L (Hg^2+^)	[66]
Gold nanoparticle	D-glutamic acid	A high-throughput, sensitive, specific, multiplex assay	1.2−125.0 nM	0.2 nM	[67]
